# Evolution Law of Helium Bubbles in Hastelloy N Alloy on Post-Irradiation Annealing Conditions

**DOI:** 10.3390/ma9100832

**Published:** 2016-10-14

**Authors:** Jie Gao, Liangman Bao, Hefei Huang, Yan Li, Jianrong Zeng, Zhe Liu, Renduo Liu, Liqun Shi

**Affiliations:** 1 Shanghai Institute of Applied Physics, Chinese Academy of Science (CAS), Shanghai 201800, China; gaojie@sinap.ac.cn (J.G.); baoliangman@sinap.ac.cn (L.B.); zengjianrong@sinap.ac.cn (J.Z.); liuzhe@sinap.ac.cn (Z.L.); liurenduo@sinap.ac.cn (R.L.); 2 School of Physical Sciences, University of Chinese Academy of Sciences, Beijing 100049, China; 3 Institute of Modern Physics, Fudan University, Shanghai 200433, China; Lqshi@fudan.edu.cn

**Keywords:** nuclear materials, radiation damage, helium bubble, denuded zone, Ostwald ripening, migration and coalescence

## Abstract

This work reports on the evolution law of helium bubbles in Hastelloy N alloy on post-irradiation annealing conditions. After helium ion irradiation at room temperature and subsequent annealing at 600 °C (1 h), the transmission electron microscopy (TEM) micrograph indicates the presence of helium bubbles with size of 2 nm in the depth range of 0–300 nm. As for the sample further annealed at 850 °C (5 h), on one hand, a “Denuded Zone” (0–38 nm) with rare helium bubbles forms due to the decreased helium concentration. On the other hand, the “Ripening Zone” (38–108 nm) and “Coalescence Zone” (108–350 nm) with huge differences in size and separation of helium bubbles, caused by different coarsening rates, are observed. The mechanisms of “Ostwald ripening” and “migration and coalescence”, experimentally proved in this work, may explain these observations.

## 1. Introduction

Nickel-based Hastelloy N alloy has been widely accepted as a promising structural material candidate for molten salt reactor (MSR) systems, due to their excellent corrosion resistance to fluoride salts and high-temperature mechanical properties [1,2]. However, the irradiation-induced void swelling or helium embrittlement caused by helium bubbles will affect their service performances [3]. In order to ensure the safe operation of MSR, it is of particular importance to clarify the helium bubble evolution caused by the diffusion of helium atoms during irradiation. The origin and mechanisms of the evolution of helium bubbles on reactor structural metal materials—especially their coarsening—were largely apprehended in the last thirty years [4,5,6,7]. It is worth noting that P. J. Goodhew [8] predicted that the mechanisms of “Ostwald ripening” and “migration and coalescence” may explain the coarsening of helium bubbles, according to calculations based on a growth rate equation. However, the experimental results related to Goodhew’s prediction of these two coarsening mechanisms were rarely reported in nickel-based alloys that are likely to be used in a MSR.

In this work, the evolution law of helium bubbles in Hastelloy N alloy on post-irradiation annealing conditions has been investigated. Elastic recoil detection analysis (ERDA) was used to probe the diffusion of helium atoms under different annealing conditions. In addition, the evolution of the helium bubbles was characterized using transmission electron microscopy (TEM). A series of small and large bubbles distributed in different depths were observed when annealing at 850 °C. The presence of a distinct critical depth provides obvious evidence for the operation of different mechanisms related to the coarsening of helium bubbles. Moreover, the coarsening mechanisms (rate) driven by the “Ostwald ripening” and “migration and coalescence” with the evolution of bubble pressure are also discussed.

## 2. Experimental Procedures

Hastelloy N alloy was provided by Haynes International Company with the chemical composition of Ni (bal.), 17.1 wt. % Mo, 7.1 wt. % Cr, and 4.2 wt. % Fe (Mn, Si, C, Al, Cu, total <1.5 wt. %). The bulk material was firstly cut into 1 mm-thick sheets (6 mm × 8 mm), and they were then successively mechanically polished with silicon carbide paper and diamond polishing paste. After the vibratory polishing, these sheets were then ultrasonically cleaned with a solution of 50% acetone and 50% absolute ethyl alcohol. The prepared samples were irradiated with 30 keV He^+^ ions at room temperature using a 400 kV ion implanter located at Beijing Normal University to an irradiation dose of 2 × 10^16^ ions/cm^2^. The irradiated samples were annealed at 600 °C for 1 h, and then some of them continued to be annealed at an elevated temperature of 850 °C for 5 h in a vacuum greater than 10^−4^ Pa.

The ERDA technique was used to obtain the quantitative depth profiles of helium concentration in these samples before and after annealing. The systemic error of the amounts of helium determined by the ERDA experiments was within 5%. All ERD spectra were converted to helium concentration distribution by Alegria 1.2 code [9]. The theoretical profile of helium concentration was calculated using SRIM 2008 software [10] for comparison. Moreover, the TEM technique was used to characterize the helium bubble information of the annealed samples. Cross-sectional TEM samples were prepared by gluing the two irradiated sample surfaces, applying the method of ion milling. These samples were investigated using a FEI Tecnai G2 F20 TEM, with the maximum accelerating voltage of 200 kV.

## 3. Results and Discussion

**Figure 1:**
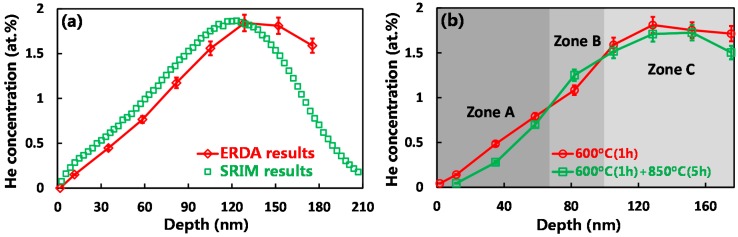
(**a**) Helium concentration depth profiles calculated using elastic recoil detection analysis (ERDA) experiments and SRIM software in Hastelloy N alloy irradiated at 2 × 10^16^ ions/cm^2^; (**b**) Evolution of helium concentration depth profiles on post-irradiation annealing conditions.

Figure 1 presents the depth profiles of helium concentration in irradiated samples before and after annealing. It is evident that there exists a discrepancy for the depth profile of helium concentration obtained, respectively, by ERDA measurement and SRIM calculation (Figure 1a). This discrepancy may be attributed to the calculation error of helium ion range using SRIM software, since temperature effects and helium mobility during the irradiation process were not considered in the SRIM calculation. The depth profiles of helium concentration of the annealed samples were measured by ERDA experiments (Figure 1b). Compared with the sample annealed at 600 °C (1 h), it is clear that the helium concentration decreased in “Zone A” (0–67 nm) after the sample was further annealed at 850 °C (5 h). It can be reasonably deduced that the release of helium atoms (bubbles) occurred near the sample surface during annealing. The diffusion or migration of helium atoms (bubbles) can explain, respectively, the increase and decrease of helium concentration in “Zone B” (67–97 nm) and “Zone C” (97–175 nm).

**Figure 2:**
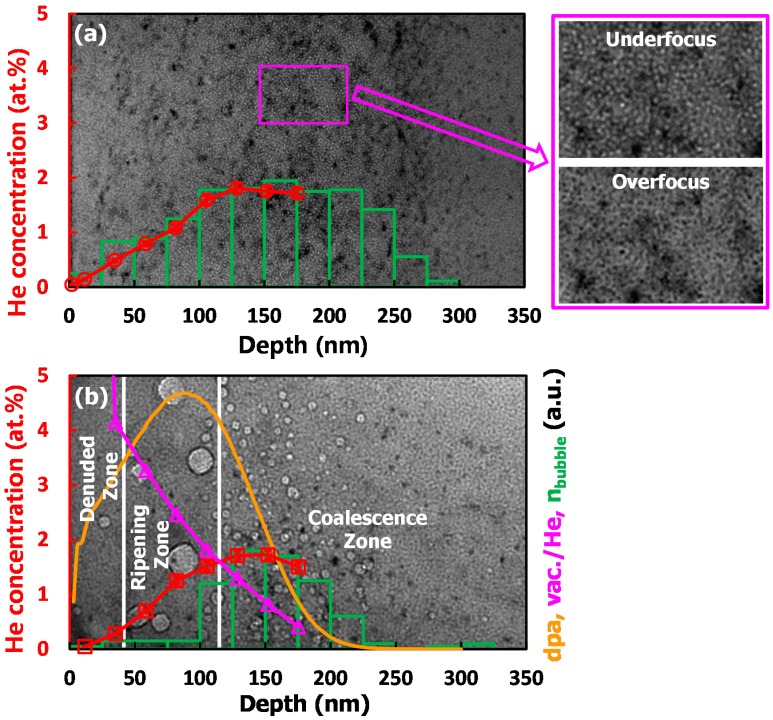
Transmission electron microscopy (TEM) images of the helium-irradiated Hastelloy N alloy in the case of (**a**) annealing at 600 °C (1 h) and (**b**) annealing at 600 °C (1 h) + 850 °C (5 h). The profiles related to the evolution of helium concentration, normalized number of helium bubbles, damage depth profile (dpa), and ratio of vacancy to helium (vac./He) are also given in this figure.

Figure 2 shows the bright-field TEM micrographs of the cross-sectioned samples after annealing under different conditions. The corresponding helium concentration profile, normalized number of bubbles (*n*_bubble_) obtained by counting bubbles in each 25 nm width sub-region, damage depth profile (dpa), and ratio of vacancy to helium (vac./He) are also given in this figure. In the case of annealing at 600 °C (1 h), a high number density of nano-scale white dots with almost the same 2 nm size were observed in the region of 0–300 nm (Figure 2a). The micrographs under the conditions of underfocus and overfocus reveal that these white dots are helium bubbles. In addition, it is found that the *n*_bubble_ distribution is consistent with the helium concentration profile. As for the sample with further higher temperature annealing, it is worth noting that rare helium bubbles can be observed in the “Denuded Zone” (Figure 2b). The width (38 nm) that is about 1–2 bubble spacing as predicted by Cowgill [11], is within the range of “Zone A” in Figure 1b. The release of helium atoms (bubbles) from the sample surface may be responsible for this observation. Furthermore, it is generally accepted that the thermal equilibrium of bubbles will be established by sufficient thermal equilibrium vacancies when the temperature is around and above 0.4 $T_{m}$ ($T_{m}$: melting temperature, 1350 °C for the alloy) [12]. Therefore, the pressures within bubbles during annealing at 600 °C and 850 °C in this work are expected to keep at the equilibrium values P=2γ/r, where γ ($\gamma \approx 2\;J\cdot m^{-2}$ in nickel [12]) and r are the surface free energy and radius of helium bubble, respectively. In Figure 2b, helium bubbles with diameter of 6 nm (1.3 GPa) in the “Coalescence Zone” (108–350 nm) and the large helium bubbles with size up to 27 nm (0.3 GPa) in the “Ripening Zone” (38–108 nm) were observed. These bubbles originate from bubbles with small diameter of ~2 nm (Figure 2a), which are suspected in over-pressurized states (4 GPa). The curves of vac./He and dpa (Figure 2b) reveal that the over-pressurized bubbles could receive more vacancies in the “Ripening Zone” than those in the “Coalescence Zone” during annealing at 850 °C. Therefore, the over-pressurized bubbles are more likely to be relaxed in the “Ripening Zone” than those in the “Coalescence Zone”. The different pressures within bubbles would trigger the different operations of coarsening mechanisms during annealing.

**Figure 3:**
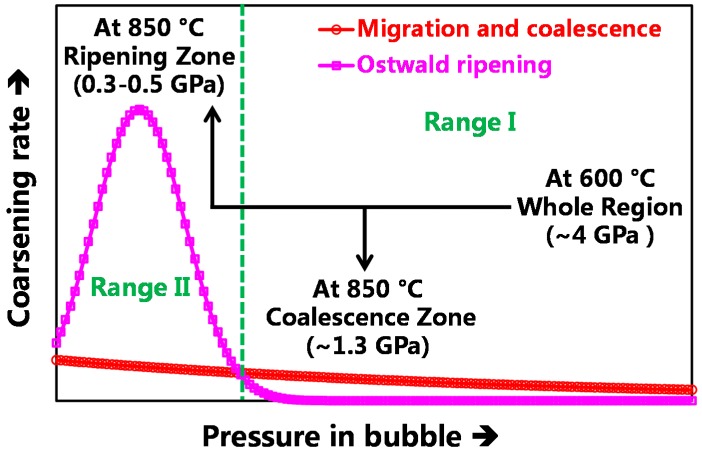
Schematic of the coarsening mechanisms (rate) driven by “Ostwald ripening” and “migration and coalescence” with the evolution of bubble pressure.

According to the “Ostwald ripening” mechanism, a decreased pressure within the bubble decreases the self-diffusion energy, which enhances the bubble migration in the matrix as well as in the bubble–matrix interface; thus, helium bubble coarsening rates will increase with decreasing pressure in bubble. As for the “migration and coalescence” mechanism, the coarsening rate will initially increase to a maximum and then decrease with decreasing pressure in bubble, since too high (too low) pressure within a bubble would increase the dissociation energy of vacancy (helium atom) dissociating from a bubble. Moreover, the calculation by P. J. Goodhew shows that the coarsening rate of “Ostwald ripening” is several orders of magnitude higher than that of “migration and coalescence” [8]. On the basis of these points, the schematic of the coarsening mechanisms (rate) driven by the “Ostwald ripening” and “migration and coalescence” with the evolution of bubble pressure can be given, as shown in Figure 3. The two curves were developed based on the assumed power law relation between the coarsening rate and the pressure within bubbles. In this work, the experimental results related to the sizes and corresponding inner-pressures of helium bubbles in different zones (Figure 2) are consistent with the helium bubbles’ evolution tendency given by the schematic. Furthermore, taking into account the huge differences in the separations and sizes of helium bubbles between the “Ripening Zone” and “Coalescence Zone”, it is believed that the coarsening driven by “Ostwald ripening” and “migration and coalescence” dominated, respectively, in the “Ripening Zone” and “Coalescence Zone”.

## 4. Conclusions

In this work, helium ion irradiation of Hastelloy N alloy with subsequent annealing was performed to investigate the evolution law of helium bubbles using ERDA and TEM. The schematic of the coarsening mechanisms (rate) with the evolution of bubble pressure was given. On this basis, the presence of a “Ripening Zone” and a “Coalescence Zone” related to the helium bubbles after high temperature annealing were believed to be mainly due to coarsening driven by “Ostwald ripening” and “migration and coalescence”, respectively. Finally, the helium bubble coarsening mechanisms based on the calculation using growth rate equation was confirmed by the experimental work in this study.
